# A Framework for Applying Global Learning to Improve Primary Health Care in the United States

**DOI:** 10.5334/aogh.3741

**Published:** 2023-02-02

**Authors:** Jonathan R. Sugarman, Alyssa K. Reed

**Affiliations:** 1 Global to Local, SeaTac, Washington, US; 2 University of Washington, Departments of Global Health and Family Medicine, Seattle, Washington, US

**Keywords:** Global health, community engagement, community health workers, primary health care, reciprocal innovation

## Abstract

**Background and objectives:**

Lessons from global health have long informed efforts to improve primary health care (PHC) in the United States (US). Despite this history, no generalizable framework exists to guide US stakeholders in the identification and application of ideas from abroad related to the key PHC components of community engagement and service delivery. We sought to develop such a framework.

**Methods:**

We reviewed the experience of Global to Local, a community-based organization (CBO) founded with a mission to apply global health strategies to improve the health in vulnerable populations in the US, and examined the experience of care delivery organizations in the US that have successfully implemented global-to-local solutions. Based on that experience, and supported by the advice of an expert panel, we developed a framework for applying global learning to improve US PHC.

**Findings:**

The framework includes six change concepts under three broad categories. The first category focuses on the need to actively and intentionally incorporate a global perspective in organizational program design and improvement activities. The second category addresses approaches to identifying global solutions related to community engagement and to health service delivery. The third category focuses on adaptation and implementation of lessons from global health in domestic contexts by applying relevant insights from dissemination and implementation science and diffusion of innovation theory.

**Conclusions:**

In the absence of a robust literature providing implementation guidance to US health systems and CBOs open to adopting or adapting PHC strategies and practices from other countries, the proposed framework synthesizing the experience of organizations that have done so can inform efforts to apply lessons from global health to improve PHC in the US.

## Background

High performing health systems throughout the world are based on a strong foundation of primary health care (PHC), and lessons from across the globe have long contributed to improved health care and health equity in the US. For instance, the establishment of the Federally Qualified Health Center program in the 1960s was heavily influenced by approaches developed in South Africa and Israel, which were in turn influenced by earlier work in China [1]. But despite the influence of global learning in the development of US health centers, there has been little continuing attention to seeking out and applying approaches from other countries to improve PHC in the US.

Recognition of gaps in performance of the US health care system relative to other countries in responding to the COVID-19 pandemic has stimulated increased interest in learning from abroad [2,3]. We use the term “global learning” to describe the process of informing local efforts to improve health, health services delivery, and health equity in the US and other high-income countries (HICs) by considering experiences and knowledge from across the globe. There is a growing understanding that adoption of innovations developed in low- and middle-income countries (LMICs) can yield effective solutions to problems faced by health systems in high-income countries (HICs) such as the US. However, there is little systematic guidance to support health care delivery organizations or community-based organizations (CBOs) in identifying or implementing such innovations to improve PHC.

We sought to develop a framework for incorporating insights from other countries into ongoing efforts of health care delivery organizations and community-based organizations to improve health outcomes and health equity. “Global Learning for US Primary Healthcare” comprises a series of change concepts, activities, and resources intended to provide guidance to US organizations that are open to global learning, but that have limited expertise and experience in global health.

## Methods

We reviewed the experience of Global to Local (G2L), a CBO located in South King County, Washington, as a starting point in developing the framework. G2L’s mission is to advance health equity and improve health in US communities through application of best practices from around the world. G2L was established in 2010 by a multi-sectoral partnership to explore the extent to which lessons from global health could be applied to improve health among communities experiencing health disparities. Inspired by the examples of innovative, and often low-cost, approaches to improving individual and community health in low-resourced environments around the world, experts in global health, local public health, and health care delivery teamed with community leaders to identify opportunities for collaboration [4]. Over the course of a decade, G2L developed programs tailored to two culturally diverse, economically disadvantaged communities- SeaTac and Tukwila-- in south King County, Washington. G2L operates community health worker (CHW) programs that help people navigate the health system, a Food Innovation Network that provides access to healthy foods and hosts an incubator program for food businesses owned by immigrant and refugee women, and a Connection Desk that connects clients with health and human services. The review included interviews with G2L staff and founding Board members, and study of archival material documenting the formation of G2L and development of its programs. We identified key elements of the process G2L used to develop its programs, with a particular focus on application of global health strategies, and developed a list of key steps that facilitated the application of global learning.

During its first decade, G2L networked with a number of US organizations and institutions that had adopted or adapted lessons from global health. To supplement G2L’s experience, we incorporated input from discussions with other US implementers identified through that networking. We then studied peer-reviewed and grey literature that addressed application of ideas from global health to improve PHC in the US.

We convened a panel of experts in primary care and global health to review an initial draft of the global learning framework during a one-day in-person meeting and sought advice on refining the framework for general use. At a subsequent virtual meeting and through desk reviews, members of the expert panel responded to a revised framework draft and implementation guidance. Based on the feedback, we modified the framework and the implementation guidance. The guide, along with a self-reflection tool and a list of activities aimed at guiding organizations to identify and act upon opportunities to operationalize concepts described in the framework, can be found at https://www.globaltolocal.org/wp/wp-content/uploads/2021/10/Global-Learning-for-US-PHC.pdf [5].

## Findings

**Figure 1:**
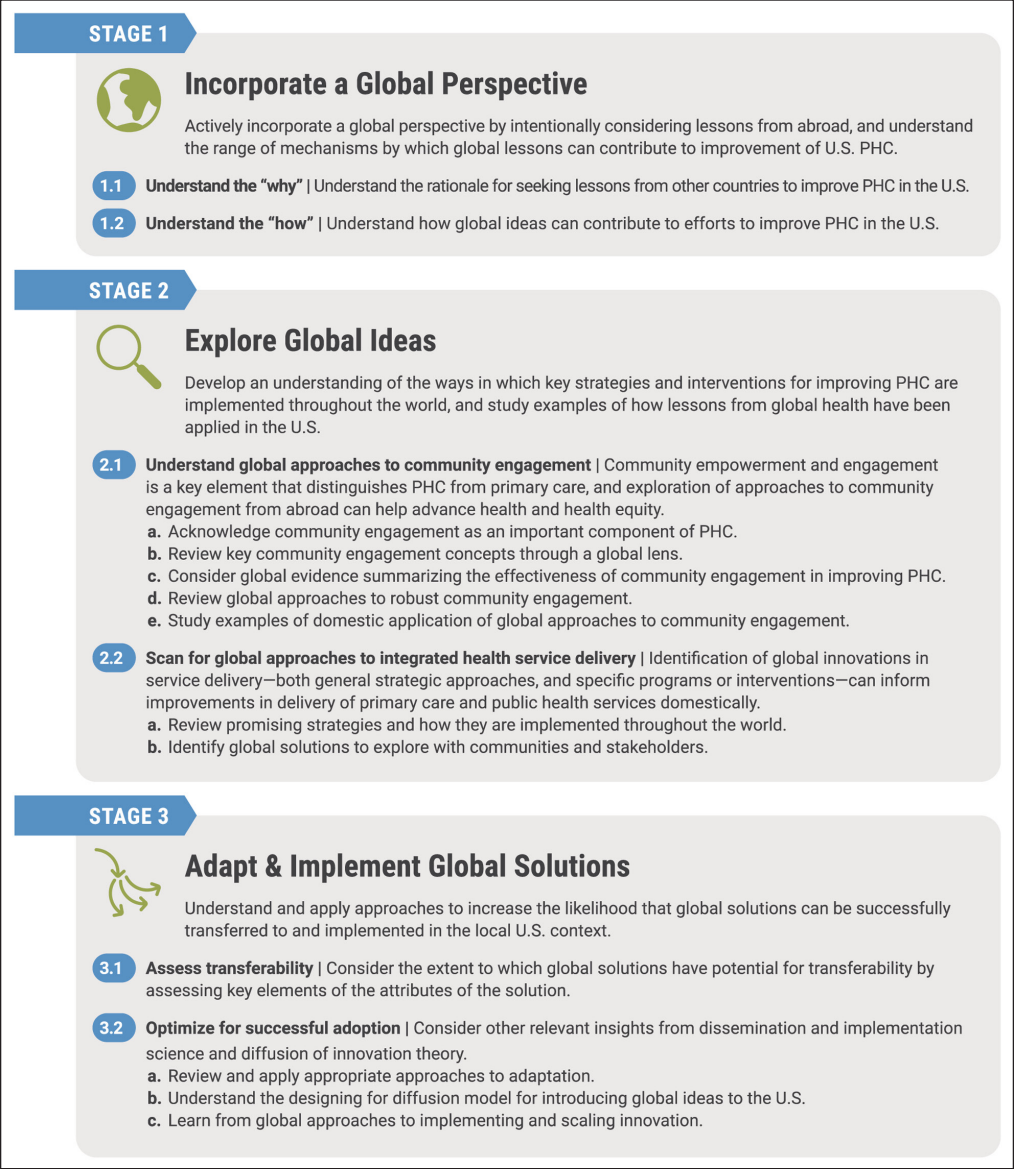
The Global Learning for U.S. Primary Health Care Framework.

The framework includes six change concepts divided among three broad categories. An overview of the framework in shown in Figure 1.

### Stage 1. Incorporate a global perspective

The first stage of the framework focuses on cultivating the intention to seek out global ideas and to incorporate them into efforts to improve organizational programs. While passive or coincidental exposure to global ideas certainly occurs, the likelihood of identifying helpful ideas from abroad can be significantly enhanced by actively committing to incorporate a global perspective into efforts to improve PHC. Change concepts in this stage address the value of understanding the rationale for global learning, and consideration of the ways in which global learning can contribute to efforts to improve PHC in the US.

#### 1.1 Understand the “why”: Understand the rationale for seeking lessons from other countries to improve primary health care in the US

Despite having a general awareness that other countries experience better health outcomes at lower cost than the US, many stakeholders in US health care have not actively considered the extent to which their work might benefit from global learning. Even in the absence of deeply ingrained provincialism or explicit rejection of the notion that ideas from other countries, particularly LMICs, hold potential for informing health system design and operation in the US, it may be difficult for some stakeholders to envision why incorporation of ideas from other nations might add value to their work. Understanding the extent to which common PHC strategies in the US such as the deployment of CHWs were modeled on approaches developed in other countries may provide motivation for identification of other globally-developed solutions to local problems.

#### 1.2 Understand the “how”: Understand how global ideas can contribute to efforts to improve PHC in the US

Contextual differences in domains such as health care financing and delivery system design, particularly in LMICs, may make it difficult to accept that solutions from other countries can be applied in the US, particularly if approached with the assumption that such solutions require a similar environment in order to achieve similar outcomes. But examples from global health can influence the work of organizations in the US in several ways. In some cases, US implementers may consider adopting or adapting a specific program, product, or tactic that was developed in another country for local use. Typically, the specific intervention is a creative innovation that addresses a problem for which existing domestic solutions are not working well, or have not been identified. A second way in which global ideas can contribute to domestic efforts is through application of a general strategy or approach drawn from another nation or group of nations, but that is modified for use in significant ways when implemented in the US. Finally, even when specific interventions, clinical programs, or approaches from other countries are not directly transferable to a local problem, creative solutions--particularly those developed with limited resources that have overcome formidable barriers-- can provide inspiration to US implementers. Examples of the how US implementers have applied adapted specific programs, applied general strategies, or have been inspired to design local solutions based on exposure to other health systems are shown in Table 1.

**Table 1:** Pathways by which Global Ideas Can Lead to Local Solutions: Examples of Specific Programs, General Strategies, and Inspiration.


GLOBAL EXAMPLE	U.S. ADOPTION OR ADAPTATION

**Specific Program:** The Friendship Bench is a program developed in Zimbabwe that trains lay workers to deliver care to people with mild to moderate behavioral health disorders. A primary driver for the development of the Friendship Bench was lack of access to professional mental health workers—a circumstance present in many US communities as well. In Zimbabwe, the lay workers are primarily grandmothers who deliver a talk therapy intervention customized for the local language and culture.	The Friendship Bench model was adapted for implementation by peer counselors and CHWs in New York City as part of ThriveNYC, a city-sponsored mental health initiative [36].

**General Strategy:** The “accompaniment” model, developed and used in several LMICs, combines clinical care and social support delivered through CHWs (*accompagnateurs* in Haiti and Rwanda; *acompañantes* in Mexico)[37]	Partners in Health’s Prevention and Access to Care and Treatment (PACT) program applied the accompaniment model to address medication adherence, health education, healthcare navigation, and access to behavioral health services to people with HIV/AIDS in Boston [38,39].

**Inspiration:** Cuba’s primary care system includes neighborhood *consultorios*—clinics staffed by nurses and physicians living adjacent to the clinics and among the patient population —which allow a deep connection between health care providers and the neighborhood they serve, contributing to highly effective patient- and community-centered care [40].	A delegation of New Mexico healthcare providers and community members focused on improving the health of residents of Albuquerque’s South Valley traveled to Havana on a trip to better understand the Cuban healthcare system. Inspired by the community connectedness of the *consultorios*, but unable to duplicate the model directly, the South Valley Community Partnership for Health Equity started a weekly walking group in which health professionals gained a greater understanding of community needs by joining residents for hour long walks around neighborhoods surrounding the clinic [41].


### Stage 2. Explore global ideas

The second category encourages development of an understanding of the ways in which key strategies and interventions for improving PHC are implemented throughout the world. Key changes in this category address identification of lessons from global health that can inform domestic efforts to enhance community engagement and improve service delivery.

#### 2.1 Understand global approaches to community engagement

Although most health care delivery organizations and CBOs in the US strive to engage the communities they serve to some degree, the PHC model as envisioned by the Alma Ata and Astana declarations has not been broadly adopted in the US [6]. While there is a large US literature addressing community engagement, there is much to learn from methods and priority given to community engagement implemented in other countries. The framework highlights five activities to support incorporation of global learning regarding community engagement into local work:

- Acknowledge community engagement as an important component of PHC
- Review key community engagement concepts through a global lens
- Consider global evidence summarizing the effectiveness of community engagement in improving PHC
- Review global approaches to robust community engagement
- Study examples of domestic application of global approaches to community engagement

The opportunity to enhance attention to the critical PHC component of community engagement and empowerment is increasingly recognized as a critical strategy for improving population health, and particularly for addressing racial and ethnic disparities in care [7]. Acknowledgment (2.1a) of the importance of community engagement in program planning, implementation, and evaluation can be formally documented in strategic plans and quality improvement processes. US implementers can explore global perspectives regarding how concepts (2.1b) such as social justice versus utilitarian motivations for engaging communities [8], or the importance of incorporation of community voices in identifying and defining problems [9], can influence health initiatives. Systematic reviews and meta-analyses of the impact of community engagement among disadvantaged populations in the Global South and Global North have highlighted its effectiveness (2.1c) in improving a variety of health-related outcomes and behaviors [10,11,12]. A recent study of approaches to community engagement (2.1d) in local health systems in thirteen countries highlighted a number of tools and practical examples of ways in which US implementers working to enhance PHC can mobilize communities to participate in improvement of health and health services [13].

US organizations have applied global approaches to community engagement in a variety of US settings. Study of the domestic application (2.1e) of approaches from elsewhere, particularly from LMICs, demonstrates “proof of concept” that global approaches to community engagement can be successfully adopted or adapted in the US. For instance, Baltimore CONNECT, a partnership between the Johns Hopkins health system and CBOs, was heavily influenced by the WHO African Partnerships for Patient Safety Community Engagement model developed in sub-Saharan Africa [14,15,16]. Other examples have been generated by MEDICC, a US based nonprofit organization that promotes learning exchanges between US and Cuban community health leaders. MEDICC’s Community Partners for Health Equity program organizes tours in Cuba for US community residents and leaders affected by health disparities in order to glean insights from the Cuban approach to health [17]. Community tours in Cuba have inspired expansion of CHW programs in the Bronx and development of health promotion efforts such as gardening, cooking, and fitness classes in New Orleans [18].

#### 2.2 Scan for global approaches to integrated health service delivery: Identification of global innovations in service delivery- both general strategic approaches, and specific programs or interventions—can inform improvements in delivery of primary care and public health services domestically

##### a. Review promising strategies and how they are implemented across the world

Early in its development, G2L commissioned PATH, a global health innovation organization, to conduct a landscape assessment to identify general global health strategies that could be transferable to the US. The assessment, first conducted in 2010, and then updated in 2016, resulted in identification of 11 global strategies such as use of community health workers, linkage of primary care and public health, and promotion of community asset building through community based organization. PATH ranked each strategiy on its transferability and feasibility in US settings, overall effectiveness, cost-effectiveness, ability to reduce health disparities, and ability to address the social determinants of health [19].

Although none of the strategies are unique to other nations, the emphasis they receive and the manner in which they have been applied may inform US implementers as they strive to improve current programs or work to develop new ones. Thoughtful review of the applicability of these strategies and others such as task shifting [20] that are commonly used in LMICs may help US implementers identify innovative solutions to a broad range of health-related challenges confronted by health care delivery systems and health and human services CBOs.

##### b. Identify global solutions to explore with communities and stakeholders

While literature searches of standard databases can assist US implementers in identifying promising innovations from abroad in order to scan for potential solutions to local challenges, other mechanisms exist by which such ideas can be identified. A number of specialized websites and databases, some of which are shown in Table 2, catalog global health innovations that may be difficult to identify through standard literature searches.

**Table 2:** Selected Web-based Resources to Scan for Global Health Innovations Potentially Transferable to the US.


RESOURCE	DESCRIPTION	NAVIGATION TIPS

Consortium of Universities for Global Health: Link Library [42].	A compendium of >180 global health-related websites. The content resulted from a Google search of the term “global health” that vetted ~18 million hits down to > 180 sites.	The “link library” divides content into four types: (1) informational resources; (2) journals and ports with high relevance to global health; (3) job and field placement opportunities; and (4) language training programs. Click on a hyperlink to go directly to that webpage.

Primary Health Care Performance Initiative (PHCPI): Improvement Strategies [43].	PHCPI developed an interactive tool that comprises modules essential for strong PHC systems, inputs, and service delivery. Drawing on evidence-informed strategies globally, each module includes an evidence review, case studies, key questions, and infographics to guide selection of strategies.	PHC improvement factors are divided by type to facilitate easy browsing. Resources include infographics, PowerPoints, downloadable PDFs and peer-to-peer learning opportunities.

National Association of County and City Health Officials Global-to-Local Public Health Exchange [44].	The Exchange includes resources such as a blog and podcast series, white papers and reports describing how successful approaches in other countries can be adopted by US local health departments.	Guidance tools are divided into three categories: “implementation”; “success stories” and “resources”. Scroll through these tabs to identify relevant tools.

Global Innovation Exchange [45].	This open-source database highlights over 5,000 examples of innovations in 13 health focus areas from over 135 low-and-middle-income countries.	Search innovations by implementation location, focus areas (sector or topic), stage (ideation through sustained scale), funder type, and recognizing organizations.

Center for Health Market Innovations (CHMI) Database of Programs [46].	The CHMI public database provides information on 1,400 innovative health enterprises, nonprofits, public-private partnerships, and policies in low- and middle-income countries that are advancing health care quality and affordability.	Browse the database for health innovations by health focus, approach, country or theme.

Innovations in Healthcare (IhI) [47].	As of 2021, the network included 90+ innovators in nearly 90 countries, with primary goals of sourcing, strengthening, scaling and studying innovations in healthcare.	Search the database by country of origin, target population, target income level, geographic reach, target settings, health need, continuum of care, offering, and organization type.

Grand Challenges Canada [48].	Lists over 1,300 innovations in 106 countries aiming to accelerate the achievement of the United Nation’s Sustainable Development Goals, with a particular focus on seven areas having the greatest potential for impact using innovation: maternal, newborn and child health; early childhood development; mental health; safe abortion; sexual and reproductive health; sanitation; and gender equality.	This database includes a search engine that filters by geographic region, program, institution, priorities and platforms.

Global Ideas for U.S. Solutions[49].	The webpage includes links to descriptions of initiatives from abroad that can be applied to advance health and health equity in the US.	Users can filter and scan resources by topic or content type.


In our review of successful examples of US application of ideas from abroad, we noted that in many cases US implementers either had previous international experience as health care providers, had participated in programs such as the Peace Corps, or were otherwise associated with academic institutions or non-governmental organizations (NGOs) with a global health focus. A growing number of health care professionals with training and experience in global health are employed in US health systems and CBOs, and represent a significant source of expertise that can be applied domestically.

Health professional as well as non-health professional members of immigrant and refugee communities can contribute well-informed and sophisticated insights about successful approaches to improving health and health care in the US. Collaboration between health professionals and immigrant community members with lived experience from other cultures and health systems can yield important improvements in care delivered to specific cultural populations. CBOs can serve as facilitators and intermediaries in order to engage community members in the co-design of culturally-congruent programs informed by knowledge and experience from their cultures and native countries. For instance, the Mama Amaan [21] project in Seattle was a collaboration led by Somali women researchers and practitioners that brought together partners such as the Somali Health Board (a CBO comprised of Somali Health professionals and volunteers working to reduce health disparities among immigrants and refugees), Health Alliance International (a global health NGO), Somali Doulas Northwest (a provider of doula services to low-income, refugee, and immigrant women; now known as Global Perinatal Services), and University of Washington faculty to support improved perinatal experience and outcomes for local Somali families. While not explicitly focused on global learning *per se*, the project was informed by global wisdom shared among the participants.

### Stage 3. Adapt and implement global solutions

The third stage of the framework focuses on adaptation and implementation of lessons from global health in domestic contexts. Change concepts in this category address assessment of the likelihood of successful transfer of a global innovation, as well as consideration of insights from dissemination and implementation science and diffusion of innovation theory that are potentially relevant to US adoption or adaptation of global solutions.

#### 3.1 Assess transferability: Consider the extent to which global solutions have potential for transferability by assessing key elements of the attributes of the solution

A number of approaches exist to assess likelihood of successful transfer of interventions from one setting to another. While some of these general approaches have been applied to assess the transfer of interventions from from one country to another, they have not always been found to be particularly useful for that purpose [22]. One approach specifically designed to assess potential transferability from LMICs to HICs was developed by Bhattacharya and colleagues [23]. The brief two-stage screening tool based on the Bhattacharya criteria was used successfully by the Henry Ford Health System to identify candidate interventions from LMICs to adopt in Detroit [5, p. 36].

#### 3.2 Optimize for successful adoption: Consider other relevant insights from dissemination and implementation science and diffusion of innovation theory

##### a. Review and apply appropriate approaches to adaptation

A number of resources, ranging from relatively simple high-level guidelines to complex frameworks intended to support rigorous implementation research, have been proposed to aid efforts to translate interventions to new environments [24,25,26,27].

However, with few exceptions, the robust literature concerning the replication, adaptation, and adoption of health programs across settings has not focused on transnational transfer of interventions or programs. Despite this fact, adaptation models that were not explicitly developed for the purpose of facilitating transfer of programs across international borders have been creatively applied to such efforts. For instance, Ogbolu and colleagues used Stirman’s adaptation model [28] to support modifications to Saúde Criança, a Brazilian family-centered innovation to address social isolation, for application among socioeconomically vulnerable families in Baltimore. Working with the Brazilian originators of the program, Ogbolu’s team identified core elements of the program, as well as elements that required modification due to context and cultural differences. The team reported that systematic application of the Stirman model supported preservation of fidelity to the core elements of the intervention, while permitting adaptations to make the intervention appropriate for the Baltimore context [29].

##### b. Understand the Designing for Diffusion model for introducing global ideas to the US

Diffusion of innovation theory seeks to explain the spread of ideas across locations or within social systems. Insights from diffusion of innovation research and practice paradigm can inform efforts to adopt ideas from abroad to the US. Dearing and colleagues applied insights from diffusion of innovation research and practice paradigm to develop a useful model to explain factors that can facilitate effective introduction of global ideas and health innovations to the US [30]. A key objective of the model is to describe how to increase the likelihood that a global innovation is “noticed, positively perceived, accessed and tried, and then adopted, implemented, and sustained in particular practice settings” through a process known as Designing for Diffusion (D4D) [31]. D4D is distinguished from general diffusion of innovation theory based on the seminal work of Everett Rogers [32] in that D4D endeavors to affect, and not simply to describe, diffusion of innovations. Dearing’s conceptual Model for Introducing Global Ideas to the US addresses how global health innovations reach the US, identifies factors that stimulated adoption by organizations and communities, and describes scale up strategies that can support or inhibit an innovation’s spread from site to site.

##### c. Learn from global approaches to implementing and scaling innovation

Just as all health is global health, the fundamental processes of implementing, diffusing, and scaling up innovations from one setting to another are similar in domestic and international contexts. Insights from transnational adoption of health programs across the world can be applied to implementation of global innovations in the US. For instance, the AIDED Model, developed by team of researchers from the Yale Global Health Leadership Institute, follows the arc of dissemination, diffusion, and scale up activities of efficacious global health innovations [33,34]. While it does not directly address transfer of global innovations from LMICs to the US, the model may provide insights to US implementers as it provides an integrated and practical approach for introduction of innovations into new settings. A useful practitioner’s guide includes a series of guiding questions for practitioners wishing to apply the AIDED model to design, implement, and scale up global health innovations [35].

## Conclusion

Application of lessons from global health has the potential to improve PHC in the US. This potential has been realized in a number of US health systems and CBOs. But despite these examples, few resources exist to support organizations wishing to identify and apply solutions from across the globe to their own work. This framework, which synthesizes the experience of organizations that have successfully done so, can serve as a starting point to inform efforts to apply lessons from global health to advance health and improve health equity in the US.
